# Rule-based versus probabilistic selection for active surveillance using three definitions of insignificant prostate cancer

**DOI:** 10.1007/s00345-015-1628-y

**Published:** 2015-07-10

**Authors:** Lionne D. F. Venderbos, Monique J. Roobol, Chris H. Bangma, Roderick C. N. van den Bergh, Leonard P. Bokhorst, Daan Nieboer, Rebecka Godtman, Jonas Hugosson, Theodorus van der Kwast, Ewout W. Steyerberg

**Affiliations:** Department of Urology, Erasmus University Medical Center, Room Na1710, P.O. Box 2040, 3000 CA Rotterdam, The Netherlands; Department of Public Health, Erasmus University Medical Center, Rotterdam, The Netherlands; Department of Urology, Sahlgrenska Academy at Göteborg University, Göteborg, Sweden; Department of Pathology, Toronto General Hospital, University Health Network, Toronto, Canada

**Keywords:** Prostatic neoplasm, Active surveillance, Risk stratification, Selection for active surveillance, Inclusion criteria active surveillance, Nomogram

## Abstract

**Purpose:**

To study whether probabilistic selection by the use of a nomogram could improve patient selection for active surveillance (AS) compared to the various sets of rule-based AS inclusion criteria currently used.

**Methods:**

We studied Dutch and Swedish patients participating in the European Randomized study of Screening for Prostate Cancer (ERSPC). We explored which men who were initially diagnosed with cT1-2, Gleason 6 (Gleason pattern ≤3 + 3) had histopathological indolent PCa at RP [defined as pT2, Gleason pattern ≤3 and tumour volume (TV) ≤0.5 or TV ≤ 1.3 ml, and TV no part of criteria (NoTV)]. Rule-based selection was according to the Prostate cancer Research International: Active Surveillance (PRIAS), Klotz, and Johns Hopkins criteria. An existing nomogram to define probability-based selection for AS was refitted for the TV1.3 and NoTV indolent PCa definitions.

**Results:**

619 of 864 men undergoing RP had cT1-2, Gleason 6 disease at diagnosis and were analysed. Median follow-up was 8.9 years. 229 (37 %), 356 (58 %), and 410 (66 %) fulfilled the TV0.5, TV1.3, and NoTV indolent PCa criteria at RP. Discriminating between indolent and significant disease according to area under the curve (AUC) was: TV0.5: 0.658 (PRIAS), 0.523 (Klotz), 0.642 (Hopkins), 0.685 (nomogram). TV1.3: 0.630 (PRIAS), 0.550 (Klotz), 0.615 (Hopkins), 0.646 (nomogram). NoTV: 0.603 (PRIAS), 0.530 (Klotz), 0.589 (Hopkins), 0.608 (nomogram).

**Conclusions:**

The performance of a nomogram, the Johns Hopkins, and PRIAS rule-based criteria are comparable. Because the nomogram allows individual trade-offs, it could be a good alternative to rigid rule-based criteria.

**Electronic supplementary material:**

The online version of this article (doi:10.1007/s00345-015-1628-y) contains supplementary material, which is available to authorized users.

## Introduction

Early detection of prostate cancer (PCa) has led to increased prevalence of finding indolent tumours, i.e. tumours that are unlikely to become symptomatic during life. The ability to predict indolent PCa is needed to avoid overtreatment [1]. Active surveillance (AS) has emerged as a feasible strategy to decrease the overtreatment of low-risk PCa. With AS, men with low-risk PCa are strictly monitored over time, and if risk reclassification or disease progression occurs, they can opt for curative therapy. Hence, the aim of AS is to safely delay or completely avoid side effects of active therapy [2]. There are 16 unique worldwide AS cohorts which all have their highly variable own protocols [3]. So far, published results on AS study cohorts worldwide show encouraging results on biochemical recurrence (BCR) rates and disease-specific mortality [4]. Long-term effects are yet unknown. Research on how to improve the existing AS protocols is, however, needed as misclassification at diagnosis, and subsequent reclassification after one-year repeat biopsy is not uncommon [5]. For example, 28 % of men within the Prostate cancer Research International: Active Surveillance (PRIAS) study were reclassified after one or more repeat biopsies [6].

Currently, all existing AS cohorts apply relatively simple combinations of inclusion criteria for patient selection (“rule-based selection”). More refined risk stratification through a nomogram may be preferable, especially in the light of individualised medicine and shared decision-making (“probability-based selection”) [7]. We aimed to assess the performance of inclusion criteria as used in several prospective AS protocols in identifying indolent cancer at radical prostatectomy (RP) and follow-up outcomes of men who received immediate RP but were also suitable for AS. For comparison, we used a previously developed and externally validated nomogram that predicts indolent disease [8, 9]. We hypothesise that the use of probabilistic selection by the use of a nomogram that incorporates multiple patient characteristics may be better for selection.

## Materials and methods

### Patients

Men included in this study were participants in the screening arm of the European Randomized study of Screening for Prostate Cancer (ERSPC). Data cohorts of the Swedish and Dutch sections of ERSPC were combined. All men were diagnosed with screen-detected PCa and underwent RP as primary treatment. Details on both Dutch and Swedish screening protocols were previously published [10, 11].

### Methods

Men with T3-4, Gleason ≥7 PCa at diagnostic biopsy or an unknown tumour volume were excluded from this analysis, as well as men with positive lymph nodes or distant metastases at the time of diagnosis or at the time of surgery. A multiple imputation model was used to fill in missing data. We used the first imputation of a multiple imputation procedure with the impute function in SPSS software (IBM Corp. Released 2012. IBM SPSS Statistics for Windows, version 21.0. Armonk, NY: IBM Corp). A total of 936 confounder values were missing, comprising 13.5 % of all values. Filling in these values through imputation allowed us to include the 382 (44 %) patients with any missing value in the analysis. All tumour characteristics were used for the multiple imputation.

We first assessed the frequency of indolent PCa at RP according to the classic definition of pT2, tumour volume <0.5 ml (TV0.5), and pathological Gleason pattern ≤3 [12]. Men not fulfilling these criteria for indolent PCa (TV > 0.5 ml and/or pathological Gleason pattern >3) were categorised as having significant PCa.

Second, we selected men from our study cohort with low-risk PCa at diagnosis defined according to the PRIAS (T1c-T2, PSA ≤ 10 ng/ml; PSA density <0.20 ng/ml/cc, Gleason ≤3 + 3, ≤2 positive cores), Klotz (T1b-T2b; PSA ≤ 10 ng/ml; Gleason ≤6), and Johns Hopkins criteria (T1c, PSA density <0.15 ng/ml/cc, Gleason ≤6, ≤2 positive cores, ≤50 % single core involvement). The frequencies of indolent PCa at RP in these groups were studied.

Third, we explored the use of a nomogram to estimate risk for indolent PCa at RP [13]. We assessed the effect of applying various eligibility criteria for the nomogram (T1c-T2a, PSA ≤ 20 ng/ml; Gleason ≤3 + 3, ≤50 % positive cores, 20 mm PCa, 40 mm benign tissue in all cores) and of different thresholds in the predicted chance of harbouring indolent PCa (referred to as Pind) on the number of men remaining suitable for AS at diagnosis.

The classic definition of a pathologic indolent PCa (pT2, TV0.5, and Gleason pattern ≤3) might be too restrictive and not reflecting biology well [14]. Therefore, we repeated steps one to three with two updated and more recent definitions of indolent PCa: (1) pT2, tumour volume <1.3 ml (TV1.3) and Gleason pattern ≤3 + 3 [14–16]; (2) pT2, Gleason pattern ≤3 + 3 and tumour volume no part of definition (NoTV) [15]. For step three, the nomogram was refitted twice using the original data [13], to account for the adjusted definitions of an indolent PCa.

Having the availability of follow-up data, we were able to calculate BCR after RP. The criteria proposed by Freedland et al. [17] were used to define BCR, i.e. one PSA value after RP > 0.2 ng/ml. The different sets of rule-based selection criteria and Pind cut-off points were compared using the Kaplan–Meier method and the log-rank test.

We finally applied decision curve analysis (DCA) [18] to evaluate the potential clinical usefulness of rule-based selection and probability-based selection models. We estimated a net benefit (NB) for the four models by summing the benefits (true-positive indolent PCa) and subtracting the harms (false-positive indolent PCa).The harms were weighted by a factor related to the relative harm of being unjustly included on AS versus being directly curatively treated while suitable for AS. This weighting was derived from the threshold probability at which a patient would opt for AS. This threshold varies between men and urologists. Clinical practice currently uses a threshold probability of 50–70 % [19]. The interpretation of a decision curve is rather straightforward; the model with the highest NB at a particular threshold should be chosen over alternative models.

*P* values (two-sided) <0.05 were considered statistically significant. For statistical analysis, we used the Statistical Package for the Social Sciences (SPSS) version 21 (IBM Corp. Released 2012. IBM SPSS Statistics for Windows, version 21.0. Armonk, NY: IBM Corp) and R version 2.15.2 (R Foundation for Statistical Computing, Vienna, Austria).

## Results

Our study cohort consisted of 864 men of whom 619 had cT1-2, Gleason 6 disease at diagnosis and were therefore eligible for analyses. Median follow-up time after diagnosis was 8.9 years. Table 1 presents the study cohort characteristics at diagnosis and outcomes after RP. With TV0.5 cut-off, a total of 229 (37 %) tumours at RP could be defined as indolent versus 390 (63 %) as significant. When applying the TV1.3 and NoTV indolent PCa definitions, the number of indolent PCa increases to 356 (58) and 410 (66 %), respectively. Pind could be calculated for 455 (74 %) men meeting the nomogram inclusion criteria.

**Table 1:** Study cohort characteristics at diagnosis and outcomes after radical prostatectomy (*n* = 619)

*At diagnosis*		
ERSPC study centre:		
The Netherlands (*n*, %)	336	54
Sweden (*n*, %)	283	46
Follow-up (years) (median, 25–75p)	8.9	5.9–10.9
Age (years) (median, 25–75p)	62.9	60.1–66.2
Clinical disease stage (*n*, %):		
T1c	395	64
T2a	157	25
T2b	37	6
T2c	30	5
PSA (ng/ml) (median, 25–75p)	4.5	3.5–6.4
Prostate volume (cc; median, 25–75p)	35.1	28.3–45.1
PSA density (ng/ml/cc; median, 25–75p)	0.13	0.09–0.18
Total number of cores (median, 25–75p)	6	6–6
Number of positive cores (median, 25–75p)	2	1–3
Total benign tissue (mm; median, 25–75p)	67.6	56.0–78.5
Total PCa tissue (mm; median, 25–75p)	4.0	2.1–8.1
Percentage cancer per positive core (median, 25–75p)	30.5	14.7–64.3
Gleason sum:		
≤6	619	100
Prediction indolent cancer (median, 25–75p) (*n* = 455 suitable for nomogram)	60 %	40–78 %
*At radical prostatectomy*		
Tumour volume (*n*, %)		
≤0.5 cc	284	46
>0.5 cc	335	54
≤1.3 cc	497	80
>1.3 cc	122	20
Gleason sum (*n*, %)		
≤6 (no pattern 4)	468	76
≥7	140	24
Indolent cancer (tumour volume ≤0.5 cc, T2, Gleason ≤3 + 3 disease)		
Yes (*n*, %)	229	37
No (*n*, %)	390	63
Indolent cancer (tumour volume ≤1.3 cc, T2, Gleason ≤3 + 3 disease)		
Yes (*n*, %)	356	58
No (*n*, %)	263	42
Indolent cancer (T2, Gleason ≤3 + 3 disease, no tumour volume cut-off)		
Yes (*n*, %)	410	66
No (*n*, %)	209	34

*25–75p* 25–75 percentile

Table 2 presents the sensitivity, specificity, positive predictive value (PPV), and negative predictive value (NPV) for all three indolent PCa definitions (TV0.5, TV1.3, NoTV) at RP of the rule-based selection and nomogram-based selection approaches. The table also contains the effect of applying different thresholds of the nomogram calculated risk of harbouring indolent PCa, i.e. Pind.

**Table 2:** Sensitivity, specificity, and BCR for several sets of rule-based and nomogram-based AS inclusion criteria for selecting indolent PCa at RP (*n* = 619)

TV0.5	Total low-risk PCa included	Total low-risk PCa missed	% included	Indolent PCa (TV ≤ 0.5) at RP included	Indolent PCa at RP missed	Sens	Significant PCa at RP included	Significant PCa at RP missed	Spec	PPV	NPV	Total BCR (%)	BCR—indolent PCa (TV ≤ 0.5) (%)	BCR—significant PCa (TV > 0.5) (%)
Total cohort	619	–	100	229	–		390	–	–	–	–	82 (13.3)	21 (3.4)	61 (9.9)
PRIAS	354	265	57	180	49	79 %	174	216	55 %	51 %	82 %	34 (9.6)	18 (5.1)	16 (4.5)
Klotz	537	82	87	207	22	90 %	330	60	15 %	39 %	73 %	66 (12.3)	18 (3.4)	48 (8.9)
Johns Hopkins	171	448	28	104	125	45 %	67	323	82 %	61 %	72 %	9 (5.3)	8 (4.7)	1 (0.6)
*Nomogram*														
Suitable	455	164	74 %	193	36	84 %	262	128	33 %	42 %	78 %	51 (11.2)	16 (3.5)	35 (7.7)
Pind >50 %	283	336	46 %	149	80	65 %	134	256	66 %	53 %	76 %	24 (8.4)	12 (4.2)	12 (4.2)
Pind >60 %	227	392	37 %	123	106	54 %	104	286	73 %	54 %	73 %	18 (7.9)	9 (4.0)	9 (4.0)
Pind >70 %	161	458	26 %	94	135	41 %	67	323	83 %	58 %	71 %	14 (8.7)	8 (5.0)	6 (3.7)

TV1.3	Total low-risk PCa included	Total low-risk PCa missed	% included	Indolent PCa (TV ≤ 1.3) at RP included	Indolent PCa at RP missed	Sens	Significant PCa (TV > 1.3) at RP included	Significant PCa at RP missed	Spec	PPV	NPV	Total BCR (%)	BCR—indolent PCa (TV ≤ 1.3) (%)	BCR—significant PCa (TV > 1.3) (%)
Total cohort	619	–	100	356	–	–	263	–	–	–	–	82 (13.3)	30 (4.9)	52 (8.4)
PRIAS	354	265	57	243	113	68 %	111	152	58 %	69 %	57 %	34 (9.6)	21 (5.9)	13 (3.7)
Klotz	537	82	87	324	32	91 %	213	50	19 %	60 %	61 %	66 (12.3)	26 (4.8)	40 (7.5)
Johns Hopkins	171	448	28	133	223	37 %	38	225	86 %	78 %	50 %	9 (5.3)	8 (4.7)	1 (0.6)
*Nomogram (refitted for indolent PCa with TV1.3)*														
Suitable	455	164	74	281	75	79 %	174	89	34 %	62 %	54 %	51 (11.2)	22 (4.8)	29 (6.4)
Pind >50 %	312	307	50	218	138	61 %	94	169	64 %	70 %	55 %	29 (9.3)	16 (5.1)	13 (4.2)
Pind >60 %	246	373	40	173	183	49 %	73	190	72 %	70 %	51 %	17 (6.9)	10 (4.1)	7 (2.8)
Pind >70 %	179	440	29	129	227	36 %	50	213	81 %	72 %	48 %	15 (8.4)	10 (5.6)	5 (2.8)

NoTV	Total low-risk PCa included	Total low-risk PCa missed	% included	Indolent PCa (pT2, Gl 3 + 3) at RP included	Indolent PCa at RP missed	Sens	Significant PCa (pT3-4, Gl > 3 + 3) at RP included	Significant PCa at RP missed	Spec	PPV	NPV	Total BCR (%)	BCR—indolent PCa (pT2, Gl 3 + 3) (%)	BCR—significant PCa (pT3-4, Gl > 3 + 3) (%)
Total cohort	619	–	100	410	–		209	–	–	–	–	82 (13.3)	39 (6.3)	43 (7.0)
PRIAS	354	265	57	263	147	64 %	91	118	56 %	74 %	75 %	34 (9.6)	24 (6.8)	10 (2.9)
Klotz	537	82	87	364	46	89 %	173	36	17 %	68 %	44 %	66 (12.3)	32 (6.0)	34 (6.3)
Johns Hopkins	171	448	28	138	272	34 %	33	176	84 %	81 %	40 %	9 (5.3)	8 (4.7)	1 (0.6)
*Nomogram (refitted for indolent PCa without TV)*														
Suitable	455	164	74	314	96	77 %	141	68	33 %	69 %	41 %	51 (11.2)	27 (5.9)	24 (5.3)
Pind > 50 %	378	241	61	273	137	67 %	105	104	50 %	72 %	43 %	40 (10.6)	25 (6.6)	15 (4.0)
Pind > 60 %	299	320	48	226	184	55 %	73	136	65 %	76 %	43 %	30 (10.0)	20 (6.7)	10 (3.3)
Pind > 70 %	202	417	33	149	261	36 %	53	156	75 %	74 %	37 %	18 (8.9)	12 (5.9)	6 (3.0)

*Sens* sensitivity, *Spec* specificity, *PPV* positive predictive value; *NPV* negative predictive value

The area under the curve (AUC) for the TV0.5 indolent definition was 0.658 for PRIAS, 0.523 for Klotz, 0.642 for Johns Hopkins, and 0.685 for the nomogram. For the TV1.3 indolent definition, the AUC for PRIAS was 0.630, for Klotz 0.550, for Johns Hopkins 0.615, and for the refitted nomogram 0.646. For the NoTV indolent definition, the AUC for PRIAS was 0.603, for Klotz 0.530, for Johns Hopkins 0.589, and for the refitted nomogram 0.608.

Table 2 furthermore presents the number of men who experienced BCR after RP according to the three definitions of indolent disease in the different sets of rule-based criteria and the nomogram suitable cohort. A log-rank test showed that the number of men experiencing BCR do not differ statistically between the groups. However, the distribution of BCR over the indolent and significant group changes, with a rising percentage of BCR in the indolent group (TV0.5 = 3.4 %, TV1.3 = 4.9 %, NoTV = 6.3 %). We found that in ROC analysis (Appendix Fig. 1), the nomogram (TV0.5) had a slightly better sensitivity-to-specificity ratio than the PRIAS rules. The AUC for the nomogram (TV0.5) was 0.610, for PRIAS 0.584, for Klotz 0.524, for Johns Hopkins 0.615, for the refitted TV1.3 nomogram 0.595, and for the refitted NoTV nomogram 0.570.

In terms of clinical usefulness, we found that in DCA analysis (appendix Fig. 2a–c), no large differences in NB were seen for threshold probabilities 50–70 %, which are clinically most relevant.

## Discussion

In our cohort of Dutch and Swedish screen-detected PCa patients who all underwent initial RP, 37 % fulfilled the TV0.5 indolent PCa criteria at RP increasing to 58 % for the TV1.3 indolent PCa criteria and 66 % for the NoTV indolent PCa definition. More stringent rule-based AS inclusion criteria as well as stricter nomogram probability thresholds decrease the rate of misclassified tumours in a rather similar fashion, but both at the cost of a substantial number of patients no longer considered suitable for AS. The nomogram based on TV0.5 had slightly better sensitivity and specificity with respect to BCR outcome than the PRIAS and Klotz criteria. If we juxtapose the TV0.5 nomogram to the Johns Hopkins criteria, the latter performed better but at the cost of including less patients and thereby curatively treating patients that might also would have been suitable for AS.

On the basis of a Kaplan–Meier analysis (curves not shown), we cannot conclude that the use of the TV0.5 nomogram is preferred over the use of rule-based selection or vice versa. However, for BCR the TV0.5 nomogram outperformed the PRIAS and Klotz criteria. The TV0.5 nomogram, however, performed slightly worse than the Johns Hopkins criteria. If we chose a slightly lower Pind and therewith allowing more men to be included on AS, sensitivity and specificity of the TV0.5 nomogram are still acceptable. This flexibility in application is a property of using a nomogram for selection rather than a strict set of rules and desirable in the light of individualised medicine and shared decision-making.

Because the classic definition of a pathologically indolent PCa may be too restrictive [14], we also used two more updated definitions of an indolent PCa. When juxtaposing the models, the TV0.5 nomogram (AUC 0.685) was slightly better in discriminating indolent from significant PCa than the PRIAS (AUC 0.658), Johns Hopkins (AUC 0.642), and Klotz (AUC 0.523) criteria. This trend of the nomogram predicting slightly better is also seen for the refitted TV1.3 and NoTV nomograms.

Perfect patient selection for AS using either rule-based selection criteria or by applying a nomogram seems difficult at present. The AUCs illustrate that both approaches are currently suboptimal in differentiating indolent from non-indolent disease at RP in a group of men with already low-risk features at diagnosis. This is confirmed by the study of Wang et al. [20] whom in a group of 273 AS patients who underwent multiple biopsies and/or delayed RP found that nomograms designed to predict indolent tumours only have a modest ability to predict biopsy progression and any progression on either biopsy or surgery in men choosing an AS management strategy. Wang et al. furthermore concluded that in a subgroup of 58 men, none of the various nomograms were able to predict surgical progression at RP [20]. Since AS is incorporated into many guidelines (AUA, NCCN, EAU, etc.) as a viable management strategy for men with either very low-risk or low-risk PCa, it is expected that more men will elect AS as their primary therapy. The optimisation of both rule-based selection and probability-based selection is therefore warranted.

Over the past few years, magnetic resonance imaging (MRI) is emerging as a tool which may be able to more accurately determine the risk of significant disease and progression of disease over time by improving sampling through target biopsies [21]. MRI may therefore also help better select AS candidates [22]. Several studies have shown the additional value of MRI in an AS protocol [21–23]. Stamatakis et al. [22] combined MRI-based factors into a nomogram which generates a probability for confirmed AS candidacy. They found that three MRI-based factors, i.e. number of lesions, lesion suspicion, and lesion density, were associated with confirmatory biopsy outcome and reclassification. A created nomogram which uses these factors has promising predictive accuracy, according to Stamatakis et al. [22]. It could be that adding such factors to the currently existing rule-based selection criteria or the nomogram could improve sensitivity and specificity and therewith AS patient selection.

A first limitation of our study lies in the fact that men in our cohort were diagnosed with sextant biopsies. Sextant biopsy does not reflect current clinical practice anymore; nowadays, current practice relies on 8–18 core biopsies. Studies that applied more extended biopsy schemes argue that with a sextant biopsy protocol, 10–30 % of cancers are missed [24]. Several studies reported that when 8–12 cores were taken, the PCa detection rate in a clinical setting might increase [24, 25]. We validated the previously developed nomogram in multiple other populations in which more extended biopsy schemes were used. Results of these validation studies showed that the nomogram predicted indolent PCa with good discrimination, indicating that it can be broadly applied in contemporary urological practice [26, 27]. In addition, we extracted correction factors for the adjustment of the nomogram with which contemporary extended biopsy schemes can be addressed [28]. Another limitation is that follow-up time of our study cohort is too short to assess mortality outcomes and relate these to baseline selection criteria. The lack of mortality outcomes was also the reason to choose BCR as an endpoint instead. Many men with BCR, however, will never develop metastasised disease or die from PCa [29]. Thirdly, patients underwent RP in different centres in either Sweden or the Netherlands. They were operated by different surgeons using different techniques for RP, which might influence outcomes. Finally, 247 cases included in this analysis were also used in the validation and construction of the nomogram. This may lead to an overestimated performance of the nomogram and Pind. The strength of this study lies in the fact that all men were diagnosed with PCa within ERSPC (Sweden and the Netherlands), resulting in standardised pathological examination of biopsy specimens and structured data follow-up [30].

In conclusion, in our cohort of Dutch and Swedish screen-detected PCa patients who all underwent initial RP, 37 % had TV0.5 indolent PCa at RP increasing to 58 % for the TV1.3 indolent PCa criteria and 66 % for the NoTV indolent PCa definition. Performance of an ERSPC-based TV0.5 nomogram and rule-based selection by the Johns Hopkins and PRIAS criteria is comparable. Because the nomogram allows individual trade-offs, it could be a good alternative to applying rigid rule-based criteria. Furthermore, a nomogram anticipates on the continuous improvement of risk assessment by newly emerging risk criteria, including imaging modalities.

## Electronic supplementary material

Supplementary material 1 (DOCX 79 kb)

## References

[1] Bangma CH, Roobol MJ, Steyerberg EW (2009). Predictive models in diagnosing indolent cancer. Cancer.

[2] van den Bergh RC, Roemeling S, Roobol MJ, Roobol W, Schroder FH, Bangma CH (2007). Prospective validation of active surveillance in prostate cancer: the PRIAS study. Eur Urol.

[3] Ip S, Dahabreh IJ, Chung M (2011). An evidence review of active surveillance in men with localized prostate cancer. Evid Rep Technol Assess (Full Rep)..

[4] Venderbos LD, Bokhorst LP, Bangma CH, Roobol MJ (2013). Active surveillance: oncologic outcome. Curr Opin Urol.

[5] Bul M, van den Bergh RC, Rannikko A (2012). Predictors of unfavourable repeat biopsy results in men participating in a prospective active surveillance program. Eur Urol.

[6] Bul M, Zhu X, Valdagni R (2013). Active surveillance for low-risk prostate cancer worldwide: the PRIAS study. Eur Urol.

[7] Gandaglia G, Giannarini G, Suardi N, Montorsi F, Briganti A (2014). Will active surveillance for clinically localized prostate cancer survive in the era of individualized medicine?. Eur Urol.

[8] Louie-Johnsun M, Neill M, Treurnicht K, Jarmulowicz M, Eden C (2009). Final outcomes of patients with low-risk prostate cancer suitable for active surveillance but treated surgically. BJU Int.

[9] Suardi N, Capitanio U, Chun FK (2008). Currently used criteria for active surveillance in men with low-risk prostate cancer: an analysis of pathologic features. Cancer.

[10] Roobol MJ, Kirkels WJ, Schroder FH (2003). Features and preliminary results of the Dutch centre of the ERSPC (Rotterdam, the Netherlands). BJU Int.

[11] Hugosson J, Aus G, Bergdahl S (2003). Population-based screening for prostate cancer by measuring free and total serum prostate-specific antigen in Sweden. BJU Int.

[12] Epstein JI, Walsh PC, Carmichael M, Brendler CB (1994). Pathologic and clinical findings to predict tumor extent of nonpalpable (stage t1 c) prostate cancer. JAMA.

[13] Steyerberg EW, Roobol MJ, Kattan MW, van der Kwast TH, de Koning HJ, Schroder FH (2007). Prediction of indolent prostate cancer: validation and updating of a prognostic nomogram. J Urol.

[14] Wolters T, Roobol MJ, van Leeuwen PJ (2011). A critical analysis of the tumor volume threshold for clinically insignificant prostate cancer using a data set of a randomized screening trial. J Urol.

[15] Van der Kwast TH, Roobol MJ (2013). Defining the threshold for significant versus insignificant prostate cancer. Nat Rev Urol..

[16] Iremashvili V, Pelaez L, Manoharan M, Jorda M, Rosenberg DL, Soloway MS (2012). Pathologic prostate cancer characteristics in patients eligible for active surveillance: a head-to-head comparison of contemporary protocols. Eur Urol.

[17] Freedland SJ, Sutter ME, Dorey F, Aronson WJ (2003). Defining the ideal cutpoint for determining PSA recurrence after radical prostatectomy. Prostate-specific antigen. Urology..

[18] Vickers AJ, Elkin EB (2006). Decision curve analysis: a novel method for evaluating prediction models. Med Decis Mak.

[19] van Vugt HA, Roobol MJ, van der Poel HG (2012). Selecting men diagnosed with prostate cancer for active surveillance using a risk calculator: a prospective impact study. BJU Int.

[20] Wang SY, Cowan JE, Cary KC, Chan JM, Carroll PR, Cooperberg MR (2014). Limited ability of existing nomograms to predict outcomes in men undergoing active surveillance for prostate cancer. BJU Int.

[21] Moore CM, Ridout A, Emberton M (2013). The role of MRI in active surveillance of prostate cancer. Curr Opin Urol.

[22] Stamatakis L, Siddiqui MM, Nix JW (2013). Accuracy of multiparametric magnetic resonance imaging in confirming eligibility for active surveillance for men with prostate cancer. Cancer.

[23] Hoeks CM, Somford DM, van Oort IM (2014). Value of 3-T multiparametric magnetic resonance imaging and magnetic resonance-guided biopsy for early risk restratification in active surveillance of low-risk prostate cancer: a prospective multicenter cohort study. Invest Radiol.

[24] Eichler K, Hempel S, Wilby J, Myers L, Bachmann LM, Kleijnen J (2006). Diagnostic value of systematic biopsy methods in the investigation of prostate cancer: a systematic review. J Urol.

[25] Chun FK, Briganti A, Graefen M (2007). Development and external validation of an extended 10-core biopsy nomogram. Eur Urol.

[26] Dong F, Kattan MW, Steyerberg EW (2008). Validation of pretreatment nomograms for predicting indolent prostate cancer: efficacy in contemporary urological practice. J Urol.

[27] Iremashvili V, Soloway MS, Pelaez L, Rosenberg DL, Manoharan M (2013). Comparative validation of nomograms predicting clinically insignificant prostate cancer. Urology..

[28] Bul M, Delongchamps NB, Steyerberg EW (2011). Updating the prostate cancer risk indicator for contemporary biopsy schemes. Can J Urol..

[29] Kweldam CF, Wildhagen MF, Bangma CH, van Leenders GJ (2014). Disease-specific death and metastasis do not occur in patients with Gleason score ≤6 on radical prostatectomy. BJU Int.

[30] Van der Kwast T, Bubendorf L, Mazerolles C (2013). Guidelines on processing and reporting of prostate biopsies: the 2013 update of the pathology committee of the European Randomized Study of Screening for Prostate Cancer (ERSPC). Virchows Arch.

