# Supplementary material for: Rule-based versus probabilistic selection for active surveillance using three definitions of insignificant prostate cancer
Source: World J Urol. 2015 Jul 10;34:253–60. doi: 10.1007/s00345-015-1628-y (PMC4729867; doi:10.1007/s00345-015-1628-y)

Rule-based versus probabilistic selection for active surveillance using three definitions of insignificant prostate cancer.

Lionne DF Venderbos^1,2^, Monique J Roobol^1^, Chris H Bangma^1^, Roderick CN van den Bergh^1^, Leonard P Bokhorst^1^, Daan Nieboer ², Rebecka Godtman^3^, Jonas Hugosson^3^, Theodorus van der Kwast ^4^, Ewout W Steyerberg^2^.

^1 Erasmus University Medical Center, department of Urology, Rotterdam, the Netherlands
2 Erasmus University Medical Center, department of Public Health, Rotterdam, the Netherlands
3 Sahlgrenska Academy at Göteborg University, department of Urology, Göteborg, Sweden
4 Toronto General Hospital, Department of Pathology-University Health Network, Toronto, Canada^

Email corresponding author: [l.venderbos@erasmusmc.nl](mailto:l.venderbos@erasmusmc.nl)

Appendix figure 1: Receiver Operating Characteristics (ROC) curve for men that experienced BCR after RP within PRIAS, Klotz, Johns Hopkins and the nomogram.


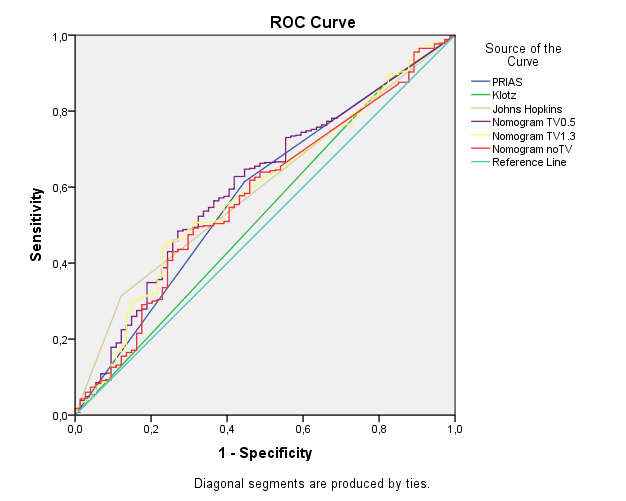


Figure 2a-c: Decision Curve Analysis (DCA) showing the ability of the PRIAS, Klotz, Johns Hopkins and nomogram to discriminate between indolent and significant disease.

A. indolent PCa at RP with TV0.5


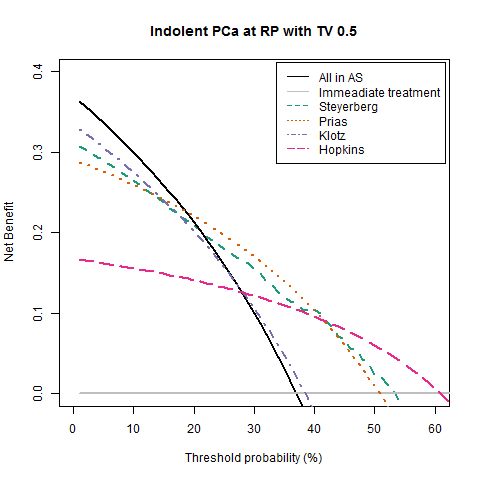


B. indolent PCa at RP with TV1.3


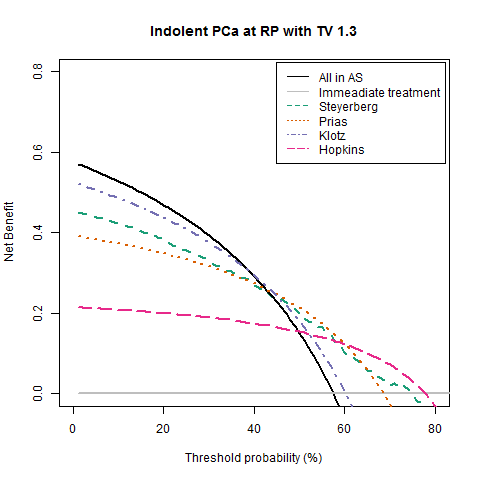


C. indolent PCa at RP with noTV


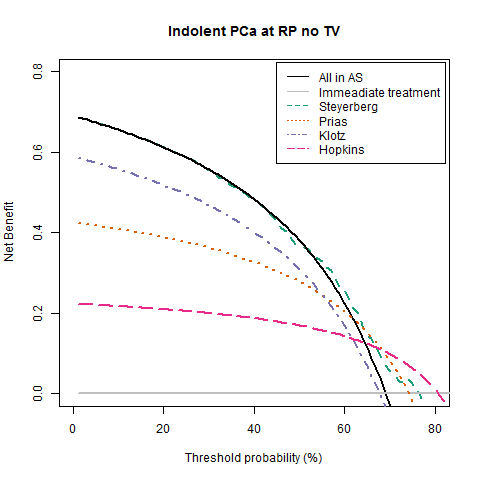

Supplement: Supplementary file 1 — Supplementary material 1 (DOCX 79 kb) [file 345_2015_1628_MOESM1_ESM.docx]
